# Supplementary material for: Lipid Metabolism Disorder in Cerebrospinal Fluid Related to Parkinson’s Disease
Source: Brain Sci. 2023 Aug 4;13(8):1166. doi: 10.3390/brainsci13081166 (PMC10452343; doi:10.3390/brainsci13081166)
Supplement: Supplementary file 1 [file brainsci-13-01166-s001.zip › brainsci-2389056-supplementary.pdf]

brain sciences

MDPI

Supplementary Materials

Supplemental Data S1. Internal standards for UPLC–MS/MS.

Compounds	CAS	Company	Catalogue Number	Concentration(μM)
LPC(12:0)	20559-18-6	Avanti	855475P	0.2
LPE(14:0)	123060-40-2	Avanti	856735P	0.2
Cer(d18:1/4:0)	74713-58-9	Avanti	860504P	0.2
MG(17:0)	5638-14-2	Sigma-Aldrich	SMB00506	0.2
PG(12:0/12:0)	322647-27-8	Avanti	840435P	0.2
PC(13:0/13:0)	71242-28-9	Avanti	850340P	0.2
PE(12:0/12:0)	59752-57-7	Avanti	850702P	0.2
DG(12:0/12:0)	60562-15-4	Avanti	800812P	0.2
TG(12:0/12:0/12:0)	538-24-9	Sigma-Aldrich	T4891	0.2
TG(17:0/17:0/17:0)	2438-40-6	Sigma-Aldrich	T2151	0.2
CE(17:0)	24365-37-5	Rhawn	R090798-1g	2
DG(17:0/17:0)	98896-81-2	Cayman	26942	0.2
FFA(16:0)-d31	39756-30-4	sigma	68277-25MG	0.2

Supplemental Data S2. Collision energy for UPLC–MS/MS.

Compounds	Class	Collision energy(V)
3-Hydroxy-dodecanoyl-carnitine	CAR	40
PC(O-18:3/20:3)	PC-O	40
PC(O-20:2/24:3)	PC-O	40
PC(14:0/18:2)	PC	40
PC(O-18:3/20:2)	PC-O	40
Cer(d18:1/24:1)	Cer	40
CE(22:6)	CE	20
CE(20:4)	CE	20
SM(d18:1/20:1)	SM	30
SM(d18:2/14:0)	SM	30
SM(d18:0/16:0)	SM	30
SM(d18:2/24:1)	SM	30
3-Hydroxy-dodecanoyl-carnitine	CAR	40

Supplemental Data S3. Expression of detected lipid families from CSF.

Lipid family	Control		PD		Adjusted p value
	Mean	Standard error	Mean	Standard error	
**SM**	4192161.61	358747.48	13301144.18	243744.23	0.000
CE	132726.40	5850.01	565730.71	14978.43	0.000
PC	31838574.32	1955571.49	69961321.68	1107961.00	0.000
Eicosanoid	23329.46	257.71	25358.91	214.06	0.482
CAR	3024803.93	23765.47	3139690.32	21416.48	0.898
MG	1886098.10	5539.85	1827825.88	15395.47	0.898
FFA	56313755.90	490044.57	57753230.47	576344.65	0.898
PS	10446.20	36.82	10332.16	56.11	0.898
TG	85585794.05	3776296.04	79958895.34	2509238.30	0.898
Cer	241959.56	4432.19	248694.19	3184.92	0.898

DG	7585293.50	20461.29	7731475.12	84025.87	0.898
PE	796128.40	41261.59	780527.94	17521.91	0.984
LPC	43964700.09	513646.12	43916878.19	338250.11	0.984

Supplemental Data S4. Expression of detected lipid species from CSF.

Lipids	Molecular weight (Da)	Control		PD		Adjusted p value
		Mean	Standard error	Mean	Standard error	
**15-oxoETE**	318.2195	19914.40	193.98	21529.47	106.81	0.136
RvD3	844.5254	3415.06	134.30	3829.44	140.40	0.882
FFA(6:0)	620.538	363573.80	15496.89	257923.71	4757.56	0.108
FFA(12:0)	620.538	696025.30	2561.26	704516.24	1099.71	0.636
FFA(14:0)	620.538	426420.20	10118.24	328388.18	7536.23	0.162
FFA(15:0)	620.538	589904.20	22123.41	441847.18	15021.21	0.363
FFA(16:0)	620.538	22444119.70	134064.46	24109442.94	217179.75	0.449
FFA(17:0)	620.538	878389.70	24189.56	763360.47	17410.40	0.622
FFA(18:0)	620.538	20642656.50	156772.49	22427468.47	176851.89	0.260
FFA(24:0)	620.538	185874.20	2960.07	150674.00	2728.75	0.140
FFA(16:1)	620.538	1250142.30	56721.00	862087.41	28370.18	0.210
FFA(17:1)	620.538	604039.50	26490.06	412850.59	16099.21	0.252
FFA(18:1)	620.538	5902023.40	126708.39	5170312.24	114879.60	0.614
FFA(19:1)	620.538	140016.80	4956.45	96867.47	3048.12	0.148
FFA(20:1)	620.538	263710.00	7683.26	218341.59	5072.49	0.449
FFA(18:2)	620.538	1484747.30	24235.71	1452857.41	33394.83	0.989
FFA(20:2)	620.538	270770.00	10799.49	191607.35	5775.18	0.195
FFA(20:4)	620.538	55969.80	1298.79	54826.47	1981.91	0.989
FFA(22:4)	620.538	54601.60	964.03	48629.71	941.10	0.614
FFA(22:6)	620.538	60771.60	2274.86	61229.06	1676.64	0.989
LPC(12:0/0:0)	620.538	1046442.00	6237.27	1043338.35	5179.70	0.989
PC(16:0/18:2)	677.4996	33812.40	1428.60	72206.65	2149.21	0.022
PS(12:0/20:3)	844.5254	10446.20	36.82	10332.16	56.11	0.917
Propionyl-carnitine	844.5254	12459.61	446.23	10684.21	187.69	0.544
Isovaleryl-carnitine	620.538	439318.70	9309.06	424237.06	3604.68	0.882
Hydroxyhexanoyl-carnitine	620.538	25709.20	207.27	25588.06	287.16	0.989
3-Methylglutaryl-carnitine	318.2195	46539.50	452.82	49918.65	413.72	0.453
3-Hydroxy-dodecanoyl-carnitine	318.2195	12650.02	1086.53	62446.65	1372.04	0.000
11-carboxyundecanoyl-carnitine	#N/A	20528.70	99.32	20398.88	132.98	0.989
3-Hydroxy-hexadecanoyl-carnitine	318.2195	44424.80	909.32	49316.65	551.52	0.467
glutaconyl-carnitine	620.538	2423173.40	13482.00	2497100.18	16299.47	0.739
CE(18:1)	650.6002	30025.92	1459.69	79019.06	1961.62	0.002
CE(18:2)	648.5845	7694.20	482.31	26882.88	848.06	0.003

CE(20:4)	672.5845	24050.08	1375.05	131180.47	4117.61	0.001
CE(22:6)	672.5845	70956.20	2844.59	328648.29	9240.39	0.000
Cer(d18:1/24:1)	647.6216	2780.54	164.37	13051.31	298.52	0.000
Cer(d18:1/26:1)	647.6216	16832.30	187.65	12798.52	287.89	0.081
HexCer(d18:1/24:1)	620.538	5110.18	314.02	18733.79	444.58	0.000
Cer(m18:1/20:0)	647.6216	23039.85	1688.10	23539.01	895.84	0.989
Cer(m18:1/22:1)	647.6216	35805.00	2745.18	31068.29	1126.54	0.882
CerP(d18:1/12:0)	647.6216	128351.30	425.80	125167.53	624.75	0.701
CerP(d18:1/16:0)	647.6216	14819.20	258.50	14035.27	251.98	0.882
CerP(d18:1/18:0)	647.6216	15221.20	190.77	10300.46	288.44	0.029
DG(12:0/20:0/0:0)	647.6216	12311.40	93.73	14351.59	167.97	0.134
DG(16:0/16:0/0:0)	568.5067	1447275.10	8005.74	1608630.12	14151.51	0.164
DG(12:0/22:0/0:0)	647.6216	17249.60	211.06	17340.65	181.35	0.989
DG(14:0/20:0/0:0)	568.5067	16169.20	228.33	18017.29	220.06	0.429
DG(16:0/18:0/0:0)	568.5067	2386262.40	12524.05	2310617.88	31375.55	0.899
DG(14:0/22:0/0:0)	568.5067	17322.80	262.19	17504.42	293.03	0.989
DG(18:0/18:0/0:0)	624.5693	2780291.60	29142.84	2751332.65	35068.67	0.989
DG(16:0/20:0/0:0)	568.5067	21661.90	346.32	20175.29	358.66	0.791
DG(14:1/16:0/0:0)	568.5067	109468.00	987.37	111795.12	821.79	0.898
DG(14:1/20:0/0:0)	568.5067	30537.80	291.91	32255.18	257.24	0.603
DG(16:0/18:1/0:0)	568.5067	37626.00	795.85	37449.47	1165.66	0.989
DG(16:1/20:0/0:0)	596.538	43118.00	234.50	41466.94	269.25	0.614
DG(16:1/22:0/0:0)	596.538	23030.00	170.30	22537.94	197.62	0.899
DG(16:0/18:2/0:0)	568.5067	18350.80	1008.19	18595.34	526.26	0.989
DG(18:1/18:1/0:0)	624.5693	75535.20	2001.52	81429.53	2814.45	0.914
DG(18:1/18:2/0:0)	624.5693	28994.50	778.16	27410.94	799.40	0.924
DG(16:1/18:3/0:0)	596.538	520089.20	6338.52	600564.76	6102.94	0.129
LPC(14:0/0:0)	620.538	28379.90	310.56	29524.88	312.89	0.844
LPC(16:0/0:0)	620.538	80122.00	4481.19	61592.24	1713.11	0.480
LPC(18:0/0:0)	620.538	139161.80	6821.01	127083.18	2965.03	0.882
LPC(20:0/0:0)	620.538	32349.70	448.61	31231.00	266.54	0.844
LPC(18:2/0:0)	620.538	62222.10	3279.49	38798.29	992.09	0.088
LPC(20:3/0:0)	620.538	18954.69	1272.71	12480.90	392.79	0.263
LPC(20:4/0:0)	620.538	40042.00	1998.34	28046.71	757.61	0.210
LPC(22:6/0:0)	620.538	51312.00	3093.95	37167.88	1172.02	0.413
LPC(O-18:0/0:0)	620.538	92630.40	1143.09	91043.29	902.84	0.952
LPC(O-20:0/0:0)	620.538	42249134.00	499589.21	42292381.18	326921.30	0.989
LPC(O-22:0/0:0)	620.538	123949.50	1691.47	124190.29	1287.43	0.989
MG(16:0/0:0/0:0)	523.3638	874385.50	3405.93	833521.06	6956.93	0.614
MG(18:0/0:0/0:0)	523.3638	1011712.60	3403.82	994304.82	9312.00	0.924
PC(16:0/14:0)	677.4996	207258.90	16921.29	632633.65	8295.06	0.000
PC(16:0/16:0)	677.4996	2229149.60	201853.91	5311162.94	87565.14	0.001
PC(16:0/18:0)	677.4996	326595.20	31872.59	677266.53	12488.55	0.013
PC(16:0/16:1)	677.4996	645252.90	38553.10	1872968.65	30676.83	0.000
PC(16:0/18:1)	677.4996	11894476.40	968813.41	30021752.	435316.48	0.000

				18		
PC(18:0/18:1)	805.5622	1475524.00	111016.37	3710983.47	68175.19	0.001
PC(14:0/18:2)	523.3638	24268.90	1651.45	110182.76	2709.69	0.000
PC(12:0/22:2)	523.3638	2982788.60	137063.45	5832130.76	179999.45	0.047
PC(18:0/18:2)	805.5622	1422498.40	108238.21	2186332.94	63968.25	0.253
PC(14:0/20:3)	523.3638	185711.10	8212.28	190042.35	4319.84	0.989
PC(16:0/20:3)	677.4996	997678.30	53332.78	1758346.35	32655.61	0.012
PC(16:1/22:2)	759.5778	583645.10	37542.49	910282.35	18014.71	0.087
PC(18:3/18:1)	831.5778	2007538.50	73669.12	3809170.47	68612.05	0.002
PC(16:0/20:4)	677.4996	5861.34	358.70	12757.85	199.26	0.001
PC(22:4/16:0)	831.5778	1844562.40	92951.64	3499647.41	51631.07	0.001
PC(18:0/22:4)	805.5622	58147.50	4179.97	136785.53	2829.69	0.002
PC(22:4/14:1)	831.5778	120165.60	9816.81	127732.88	3083.71	0.957
PC(16:0/22:5)	677.4996	355775.00	14392.62	605492.76	10970.93	0.008
PC(18:0/22:5)	805.5622	90870.90	4458.83	146322.12	2224.67	0.013
PC(16:0/22:6)	677.4996	1855743.30	100730.15	2332262.82	48596.05	0.467
PC(18:0/22:6)	805.5622	921458.50	55694.51	1287860.82	21790.26	0.164
PC(16:1/22:6)	759.5778	48935.70	2896.32	43541.29	1063.58	0.853
PC(18:1/22:6)	833.5935	235846.30	13970.78	275414.76	5483.38	0.713
PC(18:2/22:6)	831.5778	69002.20	3787.82	58107.59	1919.28	0.739
PC(O-12:0/14:0)	831.5778	69479.30	661.93	68117.76	560.69	0.913
PC(O-16:0/16:0)	831.5778	79040.60	6417.25	217225.53	3313.95	0.000
PC(O-16:0/14:1)	831.5778	7168.22	568.98	24224.71	514.36	0.000
PC(O-16:0/18:1)	831.5778	331734.80	27021.44	1286509.82	24769.18	0.000
PC(O-18:1/20:0)	719.5829	35672.70	2366.67	97229.29	2198.17	0.001
PC(O-16:0/16:2)	831.5778	29102.70	1297.23	52575.06	2087.53	0.174
PC(O-16:0/18:2)	831.5778	75774.40	4678.56	266399.06	6377.90	0.000
PC(O-16:2/18:1)	719.5829	6238.48	244.19	11235.23	896.29	0.628
PC(O-18:2/18:2)	793.5985	4215.27	137.94	11339.07	264.98	0.001
PC(O-18:3/18:2)	791.5829	141515.20	7414.08	472731.88	10249.55	0.000
PC(O-18:3/20:2)	791.5829	343020.00	27368.21	1478949.06	31308.56	0.000
PC(O-20:2/24:3)	791.5829	30730.70	2405.75	128541.59	3376.14	0.000
PC(O-18:3/20:3)	791.5829	40004.88	2796.30	157275.71	4035.31	0.000
PC(O-18:3/20:4)	791.5829	22310.03	1582.37	67580.00	1992.74	0.004
PE(16:0/18:2)	791.5829	44291.40	2049.15	53293.12	2103.40	0.780
PE(18:1/18:1)	717.5309	58905.90	3970.36	43900.24	1102.46	0.467
PE(16:0/20:4)	791.5829	62321.30	2555.36	59725.76	1794.13	0.972
PE(18:0/20:4)	717.5309	238976.30	12862.87	205039.82	4822.97	0.733
PE(18:1/20:4)	717.5309	61393.30	2749.79	68093.24	2204.34	0.882
PE(16:0/22:6)	791.5829	161065.20	9146.38	138601.71	3164.11	0.739
PE(18:0/22:6)	717.5309	126227.20	6518.86	175617.59	3965.16	0.218

PE(22:6/18:1)	763.5152	42947.80	2518.24	36256.47	1055.49	0.739
SM(d18:0/12:0)	648.5206	56694.30	511.61	47108.24	505.25	0.022
SM(d18:0/16:0)	704.5832	75059.80	6270.19	304059.18	8262.03	0.001
SM(d18:0/18:0)	732.6145	83991.47	8656.42	245227.53	4623.19	0.001
SM(d18:0/24:0)	732.6145	12176.95	638.44	41789.53	1321.33	0.003
SM(d18:1/12:0)	732.6145	1397.54	78.98	5923.12	130.43	0.000
SM(d18:1/14:0)	674.5363	55909.22	3870.44	220879.76	3661.28	0.000
SM(d18:1/16:0)	702.5676	1096841.90	90637.67	3802897.53	79583.22	0.000
SM(d18:1/18:0)	730.5989	1229017.20	124854.70	3084097.88	52212.94	0.001
SM(d18:1/20:0)	758.6302	152013.15	13091.46	491449.00	7972.41	0.000
SM(d18:1/22:0)	786.6615	171513.20	12076.75	487548.47	13018.42	0.003
SM(d18:1/24:0)	784.6458	148361.10	9484.13	471789.82	15075.67	0.005
SM(d18:2/14:0)	812.6771	199340.30	17078.11	809658.94	16058.30	0.000
SM(d18:1/16:1)	702.5676	66079.47	5911.08	233188.59	5016.80	0.000
SM(d18:1/18:1)	730.5989	240412.40	21394.62	760953.12	10712.26	0.000
SM(d18:1/20:1)	758.6302	98419.10	6458.51	414505.41	7870.48	0.000
SM(d18:1/22:1)	784.6458	94173.00	9045.70	330497.76	9364.27	0.002
SM(d18:1/24:1)	812.6771	337704.20	26595.20	1251831.59	25700.28	0.000
SM(d18:1/26:1)	812.6771	5017.81	449.92	18052.82	347.43	0.000
SM(d18:2/24:1)	810.6615	68039.50	4413.34	279685.88	7408.72	0.000
TG(14:0/14:0/16:0)	722.6424	218537.00	5315.84	176495.06	3461.43	0.218
TG(14:0/16:0/16:0)	722.6424	373027.00	7701.30	269582.88	5474.08	0.033
TG(14:0/16:0/18:0)	722.6424	105226.40	1678.08	77811.71	1403.46	0.022
TG(16:0/16:0/18:0)	792.7207	2545594.30	9353.92	2585445.18	30395.87	0.964
TG(14:0/18:0/18:0)	722.6424	1411640.10	7382.35	1412701.65	15674.91	0.991
TG(14:0/16:0/22:0)	722.6424	1231065.50	5252.76	1274526.82	11462.25	0.800
TG(14:0/18:0/20:0)	722.6424	2960673.10	10641.67	2976600.94	27562.73	0.989
TG(14:0/20:0/20:0)	722.6424	16377.90	127.37	16917.24	212.26	0.898
TG(18:0/18:0/18:0)	856.752	1459420.80	5692.13	1450975.94	12202.41	0.989
TG(12:0/16:0/16:1)	638.5485	100975.60	3568.49	60311.82	1852.47	0.027
TG(14:0/14:0/18:1)	722.6424	454539.00	14808.58	293502.18	9210.29	0.063
TG(14:0/16:0/16:1)	722.6424	810083.50	27022.95	520921.94	15953.92	0.058
TG(14:0/14:0/20:1)	722.6424	350695.70	4958.73	267673.41	6648.19	0.133
TG(14:0/16:0/18:1)	722.6424	453607.20	6960.43	309329.53	8249.80	0.029
TG(16:0/16:0/16:1)	792.7207	1478540.60	27634.20	1122050.88	31996.97	0.199
TG(16:0/16:0/18:1)	792.7207	3819503.60	136678.44	3597213.65	117042.02	0.936
TG(14:0/18:0/18:1)	722.6424	190528.60	3617.92	139726.29	3913.37	0.129
TG(14:0/16:1/20:0)	722.6424	216485.60	5732.05	144783.53	4026.24	0.047
TG(16:0/16:1/20:0)	792.7207	624552.00	22056.95	635071.18	21318.52	0.989
TG(14:0/18:1/20:0)	722.6424	497083.00	17133.48	499548.18	16763.80	0.989
TG(14:0/18:0/20:1)	722.6424	664413.70	24504.82	672777.65	21730.63	0.989

TG(16:0/16:1/22:0)	792.7207	42737.50	2318.75	37792.94	1068.77	0.844
TG(18:0/18:0/18:1)	856.752	75386.40	1792.08	66905.88	1662.15	0.723
TG(14:0/20:0/20:1)	722.6424	48736.80	2542.36	40588.18	1310.45	0.717
TG(14:1/16:0/16:1)	778.705	772624.30	28051.43	479852.41	15350.21	0.051
TG(14:0/14:1/18:1)	722.6424	71187.60	1515.65	41055.94	1075.51	0.002
TG(14:0/14:0/18:2)	722.6424	179135.40	6363.19	111836.47	3424.61	0.047
TG(14:0/16:1/18:1)	722.6424	378441.60	9504.19	265538.29	6787.08	0.064
TG(16:0/16:1/16:1)	792.7207	533612.20	11445.22	381987.76	10957.93	0.108
TG(14:0/18:1/18:1)	722.6424	2667866.00	106945.98	2402684.94	83472.66	0.882
TG(16:0/16:1/18:1)	792.7207	2694124.00	98597.45	2239899.88	71057.10	0.631
TG(14:0/18:0/18:2)	722.6424	515521.10	13385.50	428731.06	12294.24	0.557
TG(14:0/18:1/20:1)	722.6424	15289390.20	857576.22	15514278.47	543550.60	0.989
TG(16:0/16:1/20:1)	792.7207	8535278.90	498090.74	8661064.53	326390.05	0.989
TG(14:0/18:0/20:2)	722.6424	364120.00	14071.60	345506.41	10875.80	0.955
TG(16:0/16:1/22:1)	792.7207	391931.70	25424.53	397735.65	16143.07	0.989
TG(14:0/20:1/20:1)	722.6424	446420.90	29859.85	460315.00	17851.26	0.989
TG(16:1/18:1/20:0)	858.7676	1066675.10	55808.75	1113548.29	44544.20	0.989
TG(18:0/18:1/18:1)	856.752	481603.40	22961.21	511116.06	19850.62	0.964
TG(14:0/20:1/22:1)	722.6424	22191.60	1304.20	19973.90	815.23	0.909
TG(18:0/18:2/20:0)	856.752	18167.92	1108.38	16701.01	673.13	0.930
TG(18:1/18:1/20:0)	854.7363	74032.60	4372.82	61040.35	2249.93	0.739
TG(14:1/16:1/18:1)	778.705	701010.40	21482.80	475661.41	14835.47	0.102
TG(14:1/14:1/22:1)	778.705	38549.70	567.12	26511.27	710.08	0.033
TG(16:0/16:1/18:2)	792.7207	1720316.30	67961.12	1402010.82	45468.67	0.614
TG(14:0/18:1/18:2)	722.6424	387245.10	28128.47	336436.71	14462.61	0.882
TG(14:0/18:1/20:2)	722.6424	7145039.70	466417.33	6492838.82	243563.13	0.914
TG(14:0/18:2/20:1)	722.6424	2554773.40	178694.00	2409625.53	102742.92	0.985
TG(16:0/16:1/20:2)	792.7207	3348217.70	222802.83	3015967.94	120165.79	0.913
TG(18:0/18:1/18:2)	856.752	7060694.70	411717.99	6976293.53	255804.86	0.989
TG(16:0/16:0/22:3)	792.7207	202154.80	13702.45	181566.65	6788.60	0.908
TG(16:1/18:2/20:0)	858.7676	138673.70	7420.33	114765.41	3893.91	0.717
TG(14:0/20:1/20:2)	722.6424	210395.50	14796.97	180273.00	6913.77	0.853
TG(18:1/18:2/20:0)	854.7363	364035.80	23497.08	371234.94	14525.47	0.989
TG(14:0/20:1/22:2)	722.6424	248894.20	16603.64	247280.35	9638.32	0.989
TG(16:1/16:1/18:2)	858.7676	70800.50	5429.59	60571.24	2346.16	0.869
TG(14:0/18:1/20:3)	722.6424	929381.60	66151.99	776983.35	30564.68	0.818
TG(14:0/18:2/20:2)	722.6424	1138382.60	79146.87	900774.59	33715.32	0.701
TG(14:0/16:0/22:4)	722.6424	510629.10	30672.28	421696.53	14985.98	0.739
TG(18:1/18:1/18:2)	854.7363	1009448.60	52660.15	887125.35	30767.24	0.853
TG(16:0/16:1/22:3)	792.7207	79829.80	6408.09	73298.29	2887.88	0.945
TG(14:0/18:0/22:4)	722.6424	88322.90	1566.74	74021.00	1401.92	0.278

TG(14:0/20:1/20:3)	722.6424	120125.50	8406.90	104803.24	4045.61	0.882
TG(18:1/18:3/20:0)	854.7363	144100.60	9341.41	138076.47	5938.70	0.989
TG(14:0/20:2/22:2)	722.6424	50406.40	3308.18	49246.94	2002.16	0.989
TG(16:1/20:1/20:2)	858.7676	83862.10	6037.18	73881.82	2998.60	0.898
TG(18:2/18:2/20:0)	878.7363	47312.80	3747.84	42931.06	1676.53	0.924
TG(18:0/18:3/20:1)	856.752	14341.85	1182.44	13199.82	591.10	0.957
TG(16:1/16:1/20:3)	858.7676	126407.70	9357.52	95777.29	3352.47	0.614
TG(14:0/18:1/20:4)	722.6424	51908.60	2933.33	40836.24	1553.61	0.631
TG(16:0/16:1/20:4)	792.7207	43325.80	2727.85	39508.31	1431.09	0.914
TG(14:0/18:3/20:2)	722.6424	95118.10	5571.69	71634.59	2871.45	0.580
TG(18:1/18:2/18:2)	854.7363	475908.50	17715.52	388838.88	12023.73	0.594
TG(18:0/18:2/18:3)	856.752	118133.80	7591.68	97857.82	3377.84	0.739
TG(14:0/20:1/20:4)	722.6424	111045.20	7248.86	98092.76	4321.38	0.899
TG(18:1/18:3/20:1)	854.7363	60710.90	4969.31	52792.00	2521.86	0.899
TG(14:0/20:4/22:1)	722.6424	20249.28	1396.21	19738.72	796.90	0.989
TG(16:0/16:0/22:6)	792.7207	46388.70	3858.25	39986.71	1700.68	0.882
TG(18:2/18:2/18:2)	878.7363	278940.30	5612.00	208433.29	3241.65	0.022
TG(18:1/18:2/18:3)	854.7363	78976.00	3828.00	59138.24	1671.50	0.362
TG(14:0/18:3/22:3)	722.6424	54476.30	2744.48	45177.65	1212.21	0.634
TG(14:0/20:2/20:4)	722.6424	58689.60	4180.91	45099.76	1945.32	0.682
TG(14:0/20:4/22:2)	722.6424	33288.60	1897.01	30368.91	1177.24	0.914
TG(16:0/18:3/22:3)	792.7207	43005.20	3155.84	35636.31	1511.56	0.818
TG(14:0/20:1/22:5)	722.6424	58886.40	4641.59	48753.76	2398.60	0.853
TG(18:2/18:2/18:3)	878.7363	28690.70	833.05	22041.35	356.58	0.098
TG(14:0/20:2/20:5)	722.6424	57281.90	5541.82	36957.24	1623.14	0.495
TG(16:0/16:1/22:6)	792.7207	56637.30	4792.40	44108.18	1948.22	0.739
TG(16:0/18:1/22:6)	792.7207	330070.50	27211.62	264910.47	10777.98	0.773
TG(18:0/18:1/22:6)	856.752	14017.80	1030.92	13681.49	593.88	0.989
TG(14:0/20:2/22:6)	722.6424	174554.60	14803.05	126857.29	5613.08	0.631
TG(14:0/22:2/22:6)	722.6424	71490.40	5852.16	57427.71	2601.11	0.791
TG(14:0/22:3/22:6)	722.6424	38891.10	3528.34	26043.18	995.75	0.495
